# Generation of CD34^+^CD43^+^ Hematopoietic Progenitors to Induce Thymocytes from Human Pluripotent Stem Cells

**DOI:** 10.3390/cells11244046

**Published:** 2022-12-14

**Authors:** Léa Flippe, Anne Gaignerie, Céline Sérazin, Olivier Baron, Xavier Saulquin, Ignacio Anegon, Laurent David, Carole Guillonneau

**Affiliations:** 1 Nantes Université, INSERM, Center for Research in Transplantation and Translational Immunology, UMR 1064, F-44000 Nantes, France; 2 Nantes Université, CHU Nantes, Inserm, CNRS, BioCore, 44035 Nantes, France; 3 Département de Chirurgie Cardiaque Pédiatrique, CHU Nantes, 44093 Nantes, France; 4 Nantes Université, Inserm, CNRS, CRCI2NA, 44035 Nantes, France

**Keywords:** hiPSC, hESC, hematopoietic progenitor, T-cell progenitor, hematopoietic differentiation

## Abstract

Immunotherapy using primary T cells has revolutionized medical care in some pathologies in recent years, but limitations associated to challenging cell genome edition, insufficient cell number production, the use of only autologous cells, and the lack of product standardization have limited its clinical use. The alternative use of T cells generated in vitro from human pluripotent stem cells (hPSCs) offers great advantages by providing a self-renewing source of T cells that can be readily genetically modified and facilitate the use of standardized universal off-the-shelf allogeneic cell products and rapid clinical access. However, despite their potential, a better understanding of the feasibility and functionality of T cells differentiated from hPSCs is necessary before moving into clinical settings. In this study, we generated human-induced pluripotent stem cells from T cells (T-iPSCs), allowing for the preservation of already recombined TCR, with the same properties as human embryonic stem cells (hESCs). Based on these cells, we differentiated, with high efficiency, hematopoietic progenitor stem cells (HPSCs) capable of self-renewal and differentiation into any cell blood type, in addition to DN3a thymic progenitors from several T-iPSC lines. In order to better comprehend the differentiation, we analyzed the transcriptomic profiles of the different cell types and demonstrated that HPSCs differentiated from hiPSCs had very similar profiles to cord blood hematopoietic stem cells (HSCs). Furthermore, differentiated T-cell progenitors had a similar profile to thymocytes at the DN3a stage of thymic lymphopoiesis. Therefore, utilizing this approach, we were able to regenerate precursors of therapeutic human T cells in order to potentially treat a wide range of diseases.

## 1. Introduction

Immunotherapy with T-effector (Teff) or T-regulatory (Treg) cells offers new possibilities for the treatment of diseases, such as cancer or autoimmune diseases, and transplantation rejections that are in need of new therapeutics [1,2,3]. Next-generation therapeutics using Teff or Treg cells have started to emerge thanks to genetic engineering, and they present the possibility of providing antigen specificity to the genetically modified T cells by expressing TCRs or chimeric antigen receptors (CARs) in order to improve the outcome [4,5]. However, the clinical use of Teffs or Tregs has only been performed on autologous T cells, and this raises the issue of producing them in the required time window. This autologous source could also limit the number of T cells that can be produced in some instances. The development of “off-the-shelf” allogeneic T cells is awaited as the next-generation T cells that will overcome these obstacles. One approach to generating “off-the-shelf” T lymphocytes is the development of an ex vivo platform that will mimic lymphopoiesis starting with stem cells and thereby generating T cells.

Lymphopoiesis, a process resulting from the generation of progenitors from bone marrow and their maturation in the thymus, is difficult to study in humans and recapitulate in vitro. To date, the best option is a scRNAseq trajectory reconstruction, as it provides the possibility of mapping T-cell development [6]. Although hematopoietic stem cells (HSCs) can differentiate into T cells [7,8,9,10], they cannot be amplified beforehand or edited efficiently. Pluripotent stem cells are an alternative source with great potential for the production of T cells. In particular, hiPSCs reprogrammed from somatic cells are more accessible than hESCs, and they offer the opportunity to develop therapies based on antigen specificity.

Reprogramming from T cells has been shown to generate T-iPSCs with pre-existing V(D)J rearrangements at the T-cell receptor loci; in turn, using T-iPSCs could facilitate the generation of fully competent T cells. Indeed, antigenic specificity can be retained during reprogramming. It has been shown that reprogramming MART1-specific human T cells generates T-iPSCs that can subsequently differentiate into functional T-cell clones in vitro and in vivo and with the same specificity as the original cell [11,12]. Similarly, it has also been shown in mice that the adoptive transfer of CD4^+^ Tregs derived from autoantigen-specific T-iPSCs significantly reduced the CD8^+^/CD4^+^ ratio in the pancreas of diabetic mice [13]. However, the differentiation of T cells from hPSCs remains difficult, and therefore, a better understanding of the differentiation process is required.

In order to address this issue, we generated T-iPSCs, subjected those cells to T-cell differentiation and compared them with several hPSC lines. Next, we analyzed hematopoietic stem progenitor cells (HSPCs) differentiated from hPSCs vs. CD34^+^ cord blood HSCs and revealed that the CD34^+^CD43^+^ subset is the most closely related to CD34^+^ cord blood HSCs. We then subjected HPSCs from hPSCs and CD34^+^ cord blood cells to T-cell differentiation with primary thymocytes as a control in order to clearly pinpoint the phase match of differentiated cells in in vivo situations, and we demonstrated efficient differentiation up to the DN3a T-cell development stage. Therefore, our work lays down the basis for studying the establishment of TCR expression during T-cell differentiation in humans, as well as a potential method for the production of a large number of thymocytes in which engineered TCRs or CARs can be expressed.

## 2. Results

### 2.1. Efficient Establishment of hPSCs Clones from Blood T Cells

**Figure 1:**
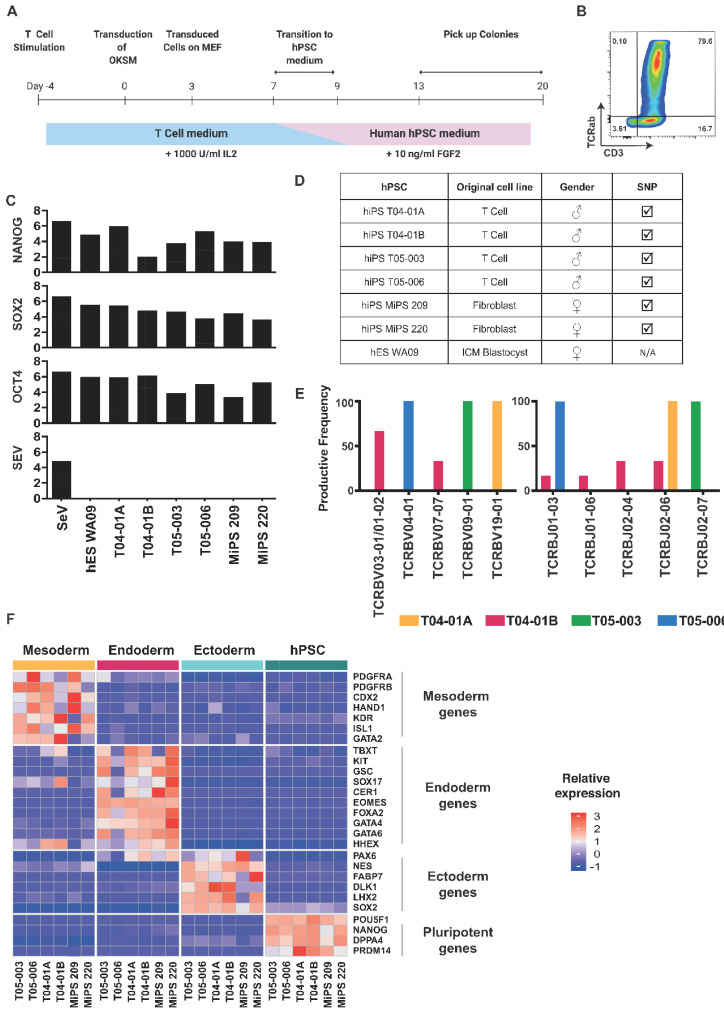
Generation of human iPSCs from T cells. (**A**) Schematic view of the reprogramming protocols. A total of 10^5^ activated T cells were seeded on day 0, then they were transduced with 3 Sendai viruses expressing polycistronic KLF4/OCT4/SOX2, MYC, and KLF4 at a ratio of 6:6:3.6, respectively. Cells were split among feeders on day 3 and placed in the hPSC media on day 9. Colonies started to appear around day 13, and they were picked on feeders. (**B**) Representative dot plot of CD3 and TCRαβ on T cells after 4 days of αCD3 and αCD28 mAbs activation. (**C**) qPCR measurement of Oct4, Nanog, Sox2, and Sendai virus (SEV) expression in the indicated cell lines, one positive control (SeV), and one negative control (hES WA09). (**D**) Summary of the original cell line, gender, and SNP profiles of the indicated cell lines. A tick mark means that it is identical to parental cells. (**E**) Analysis of TCR rearrangements in T04-01A, T04-01B, T05-003, and T05006. V, D, and J segment usages in the TCRB gene locus were sequenced and identified using an ImmunoSEQ Analyzer (Adaptive Biotechnologies). (**F**) The indicated cell lines have been differentiated in the three germ layers (mesoderm, endoderm, and ectoderm) and have been analyzed by RNAseq. Selected gene expressions representative of each germ layer were plotted as a heatmap.

We first reprogrammed T-cells from PBMCs from healthy volunteers in order to obtain T-iPSCs with rearranged TCR regions [11,12]. Prior to the induction of reprogramming with Sendai OSKM vectors (Figure 1A), we activated whole PBMCs for 4 days with αCD3 and αCD28 mAbs and recombinant IL-2, since somatic cell reprogramming is more favorable in cells with an active cell cycle [14], and obtained 80% TCR^+^CD3^+^ cells (Figure 1B). Two weeks after induction, clones were picked and amplified, and after 10 passages (2.5 months), the T-iPSCs were validated. All tested clones lost the Sendai vectors used for the induction of reprogramming factors and displayed NANOG, SOX2, and OCT4 expression levels similar to those of hESCs (H9) (Figure 1C). We also performed SNP analysis in order to confirm cell identity and infer copy number variations. We showed that the reprogrammed T cells harbored the same karyotype as the parental cells (Figure 1D). In order to confirm that the T-iPSCs clones (T04 and T05) were derived from mature T cells, we analyzed the TCR rearrangements by sequencing the V, D, and J segments in the T-cell receptor beta (TCRB) gene locus. The specific rearrangement pattern identified in the TCRB gene locus of the T-iPSCs confirmed their mature T-cell origins (Figure 1E). T04-01A, T05-003, and T05-006 iPSC clones had single Vβ and Jβ sequences, demonstrating their clonogenicity, whereas the T04-01B clone had multiple Vβ and Jβ sequences characteristic of a mosaic clone. Finally, in order to functionally test the T-iPSCs, we performed a trilineage assay which consisted of inducing the germ-layer engagement of the hiPSCs. Engagement with the mesoderm, endoderm, and ectoderm was evaluated by 3′seq-RNA Profiling (3′SRP) [15] (Figure 1F). This assay confirmed that the reprogrammed T cells could differentiate into the mesoderm, endoderm, and ectoderm. In conclusion, we were able to efficiently reprogram PSCs from T cells, therefore generating hiPSCs with rearranged TCRs.

### 2.2. Functionally Competent Hematopoietic Progenitor Cell Differentiation from Reprogrammed T Cells

**Figure 2:**
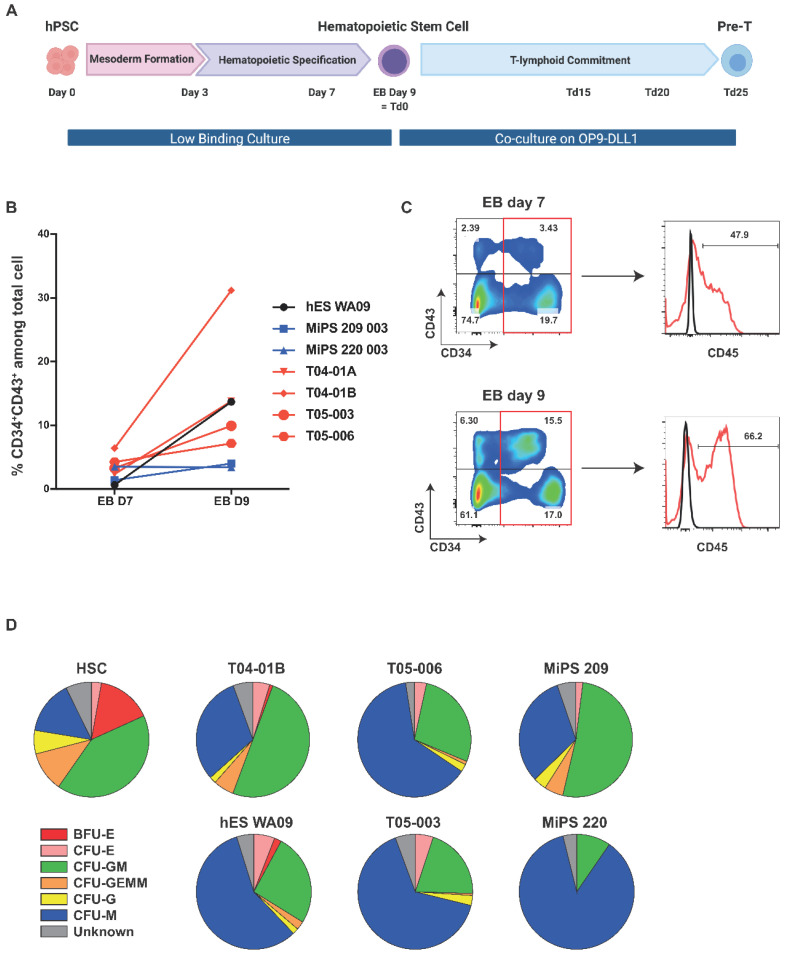
Multipotent HPSCs were derived from hPSCs. (**A**) Schematic representation of the hematopoietic stem cell differentiation protocol. Between day 0 and day 9, EBs were treated to induce firstly mesoderm formation and then to induce specification towards the hematopoietic lineage. After 9 days, the EBs were dissociated and the cells were co-cultured on OP9-DLL1 or DLL4 cells in a specific medium to induce T-lymphoid commitment. (**B**) Evolution of the percentage of total cells expressing CD34 and CD43 between day 7 and day 9 and expression of CD45. (**C**) Flow cytometry analysis of differentiating cells at D7 and D9 in EB culture from hPSC. Representative dot plots of CD34 and CD43 co-staining on living cells are shown. The expressions of CD45 in CD34^+^ cells are shown. Red line represents cells stained with a fluorescent antibody and black line represents unstained cells. (**D**) Analysis of visual microscopic counting of CFU assay blood colonies for the indicated cell lines. Data are presented as a pool of experimental duplicates, *n* = 1.

We then subjected hPSCs derived from fibroblast or T cells to HPSC differentiation to better understand their respective potential and included hESCs as controls. The differentiation was started by mesoderm induction, followed by hemogenic endothelium specification to finally yield CD34^+^CD43^+^ HPSCs on day 9 (Figure 2A). Overall, all pluripotent cell lines differentiated efficiently, yielding on average 37% of CD34^+^ cells, although we observed a more efficient generation of CD34^+^ cells using the T04.01B clone vs. the other clones (Supplementary Materials, Figure S1A). For all tested cell lines, the percentage of the CD34^+^CD43^+^ population within the CD34^+^ cells evolved drastically between day 7 (around 3%) and day 9 (around 13%) (Figure 2C). Among the CD34^+^ cells, the CD45^+^ cells increased from 40% on day 7 to 80% on day 9 (Figure 2B,C). We observed variation between the different hiPSC lines; the T04.01B clone was particularly efficient at differentiating compared to all other T cell-derived iPSC clones that behaved similarly to hESC and fibroblast-derived iPSCs. Nevertheless, studies have shown that differences in the differentiation potential are mostly due to the genetic background and gender of each parent cell line [16,17,18] (Supplementary Figure S1A,B).

To functionally assess the CD34^+^ HPSCs, we subjected them to a hematopoiesis assay on methylcellulose. Cord-blood HSC CD34^+^ cells subjected to this protocol yielded visible colonies of burst-forming unit–erythroid (BFU-E), colony-forming unit–erythroid (CFU-E), colony-forming unit–granulocyte/macrophage (CFU-GM), colony-forming unit–granulocyte/erythrocyte/macrophage/megakaryocyte (CFU-GEMM), colony-forming unit–granulocyte (CFU-G) and colony-forming unit–macrophage (CFU-M), which can be enumerated (Figure 2D and Supplementary Figure S2A).

After counting the colonies, the cells were harvested and the expression of the erythroid marker (CD235a) and monocyte/macrophage markers (CD14, CD15) were analyzed by flow cytometry (Supplementary Figure S2C) [19]. Starting from similar numbers, we found that the CD34^+^ cells derived from differentiation self-renewed less efficiently than the cord blood HSCs (Supplementary Figure S2B). The 6 hiPSCs clones tested were able to generate all types of blood colonies, albeit not in the same proportions as cord blood HSCs, as shown by morphological numeration (Figure 2D) or flow cytometry analysis (Supplementary Figure S2D). Most colonies represented macrophages and granulocytes, a recurrent result of this experiment [20,21,22,23,24]. Nevertheless, altogether, this demonstrates that the generated CD34^+^ HPSCs were functional.

### 2.3. Transcriptomic Profiling Demonstrates That CD34^+^CD43^+^ Cells Derived from hPSCs Are the Most Similar to HSCs

**Figure 3:**
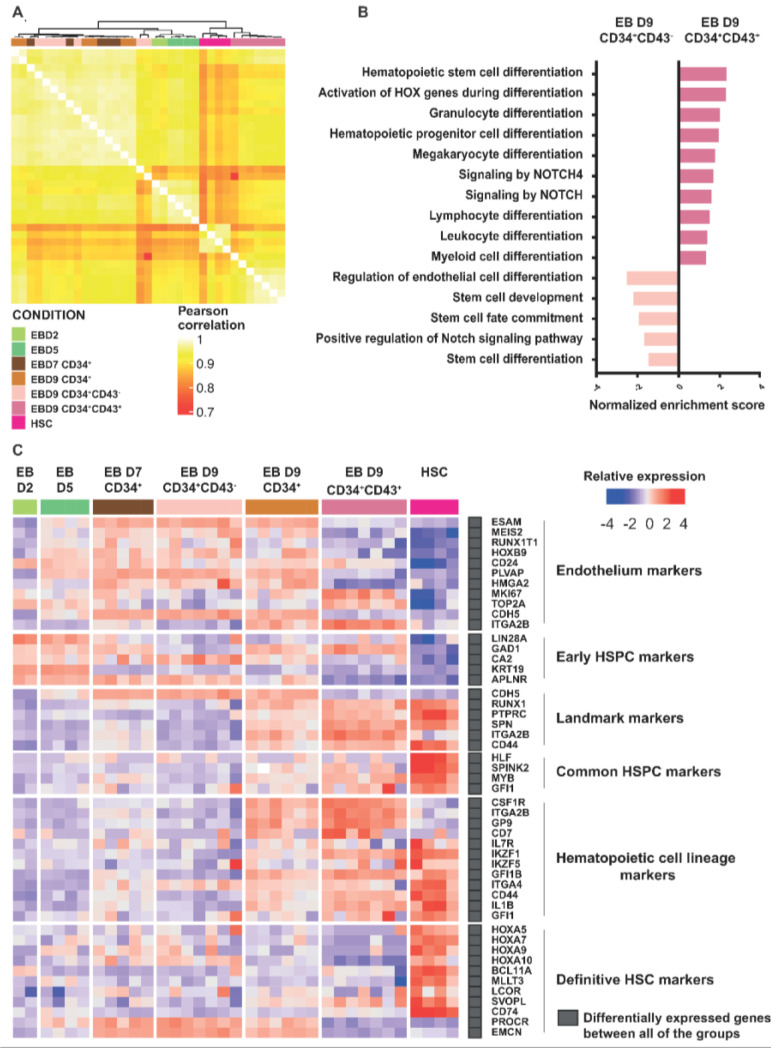
CD34^+^CD43^+^ derived from hPSCs are closely related to cord blood HSCs. (**A**) Heatmap of Pearson correlation coefficients of HSCs and hPSCs differentiated from hPSCs. (**B**) Normalized enrichment score of biological pathways upregulated or downregulated in D9 CD34^+^CD43^+^ EBs compared to HSCs. All represented pathways have an adjusted *p*-value < 0.05. (**C**) Gene expression heatmaps of selected markers characterizing hematopoietic differentiation are shown for digital gene expression sequencing (DGE-seq) for this study. The heatmap was generated by scaling and centering gene expression.

To further characterize the CD34^+^ HPSCs derived from PSCs, we performed 3′SRP RNAseq on sorted CD34^+^ and CD43^−^ cells from embryoid bodies (EBs) on day 7, CD34^−^CD43^+^, CD34^−^CD43^−^, CD34^+^CD43^+^, and CD34^+^CD43^−^ cells from EBs on day 9 (Supplementary Figure S3) and compared them to cord blood HSCs. Hierarchical clustering defined three groups of cells: (1) D7 CD34^+^, D9 CD34^+^, and D9 CD34^+^CD43^−^ EBs; (2) D2 and D5 EBs; and (3) D9 CD34^+^CD43^+^ EBs and cord blood HSCs (Figure 3A). We observed that the D9 CD34^+^CD43^+^ EBs clustered with the cord blood HSCs to form the group of HPSCs. Pearson correlation and PCA analysis further confirmed the proximity of the D9 CD34^+^CD43^+^ EBs and cord blood HSCs (Figure 3A and Supplementary Figure S4). Surprisingly, the D7 CD34^+^ and D9 CD34^+^ EBs showed a high degree of transcriptional similarity, despite relative differences in their potential to become T-cell progenitors in vitro. Functional enrichment analysis revealed that genes significantly upregulated in D9 CD34^+^CD43^+^ EB cells were involved in pathways such as hematopoietic stem cell and progenitor differentiation, the activation of HOX gene during differentiation, Notch signaling, and megakaryocyte/lymphocyte/leukocyte differentiation (Figure 3B). In comparison, genes overexpressed in D9 CD34^+^CD43^−^ EBs were linked to the regulation of endothelial cell differentiation, endothelium development, the positive regulation of the Notch signaling pathway, and stem cell development/commitment/differentiation (Figure 3B). This global analysis demonstrates that the CD34^+^CD43^+^ subset differentiated from hiPSCs was the sub-population more closely related to the cord blood HSCs, while in contrast, the CD34^+^CD43^−^ subset differentiated from hiPSCs was potentially still at the hemogenic–endothelium transition [25].

To further compare the HPSCs obtained from iPSCs and cord blood, we analyzed the expression profiles of significantly differentially expressed gene subsets associated with the progression of hematopoiesis (Figure 3C). We observed that endothelial and early HSPC markers, such as ESAM, MEIS2, HOXB9, and CD24 were upregulated in D2, D5, D7 CD34^+^, D9 CD34^+^CD43^−^, and D9 CD34^+^ EBs, while they were downregulated in D9 CD34^+^CD43^+^ EBs and HSCs. Interestingly, MEIS2, a gene involved in the endothelium-to-hematopoiesis transition (EHT) [26], was overexpressed by CD34^+^ cells at D7 and CD34^+^CD43^−^ cells at D9 and downregulated by CD34^+^CD43^+^ cells and cord blood HSCs, suggesting that the latter had undergone their EHT. On the contrary, the overexpression of the well-known markers of HSCs, such as RUNX1, PTPRC (or CD45), CD44, MYB, and IKAROS Family Zinc Finger IKZF1 (IKAROS) and IKZF5 (PEGASUS) was observed in D9 CD43^+^CD34^+^ EBs and HSCs, while they were downregulated in D2, D5, D7 CD34^+^, and D9 CD34^+^CD43^−^ EBs. Differentially expressed genes between cells show the hierarchy of acquisition of the HPSC signature. Finally, it was noted that definitive HSC markers, such as HOXA [27,28] and MLLT3 [29], which have been highlighted for their involvement in self-renewal and engraftment capacity, were upregulated in cord blood HSCs but not expressed in D9 CD34^+^CD43^+^ EBs (Figure 3C and Supplementary Figure S5). Altogether, the transcriptomic signature shows that D9 CD34^+^CD43^+^ EBs were closely related to functional CD34^+^ cord blood HSCs, and thus, represent the most promising population to support lymphopoiesis.

### 2.4. Efficient Lymphopoiesis Induction up to DN3a T-Cell Developmental Stage of HPSCs

**Figure 4:**
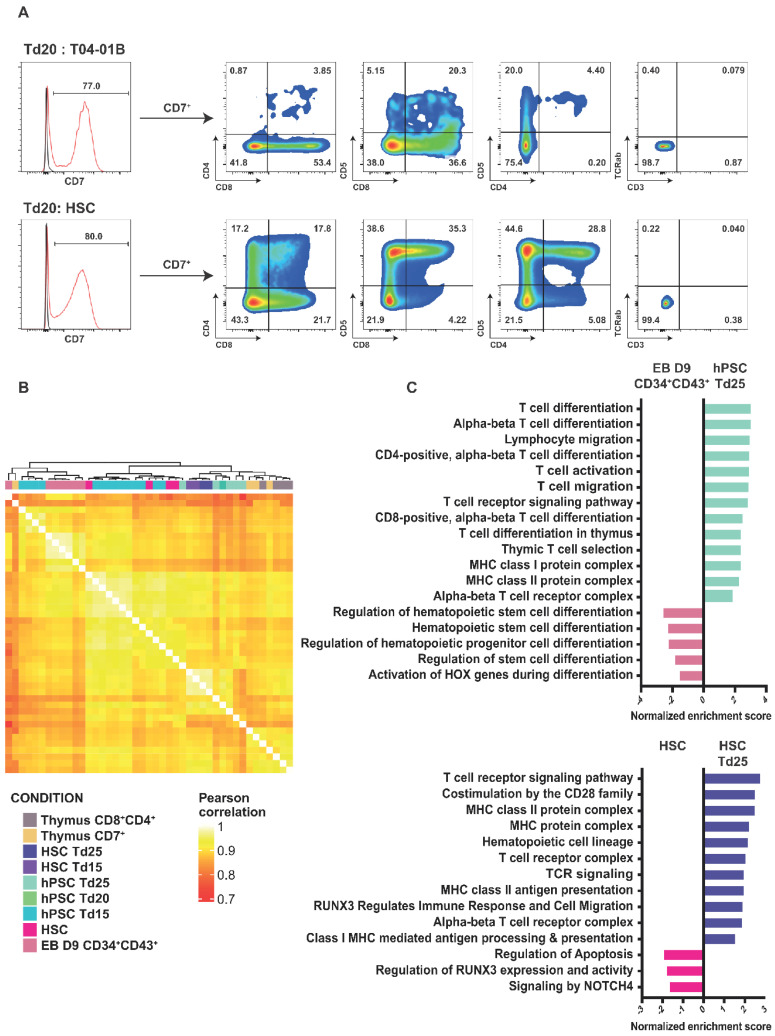
hPSCs-Td25 are closed to CD8^+^CD4^+^ thymocytes. (**A**) Representative histogram of CD7 expression in living cells (excluding OP9) on day 29. Red line represents cells stained with a fluorescent antibody and black line represents unstained cells. The expressions of CD8, CD4, CD3, and TCRαβ among CD7^+^ cells are shown in dot plot. (**B**) Heatmap of Pearson correlation coefficients of thymus cells and hPSCs differentiated from hPSCs. (**C**) Normalized enrichment score of biological pathways upregulated or downregulated in EB D9 CD34^+^CD43^+^ compared to hPSC-Td25 (up) and in HSC compared to HSC-Td26 (down). All represented pathways have an adjusted *p*-value < 0.05.

We then subjected HPSCs and CD34^+^ cord blood HSCs to T-cell differentiation using a DLL1- or DLL4-overexpressing OP9 cell co-culture. Of note, we recently defined that OP9-DLL1 and OP9-DLL4 were equivalently potent for inducing T cell lineage [30]. Briefly, D9 EBs were dissociated and seeded on OP9-DLL1/4 cells in a medium supplemented with SCF, FLT3l, and IL7 (Td0) and analyzed after 15 (Td15), 20 (Td20), and 25 days (Td25) (Figure 2A). The same treatment was applied to the CD34^+^ cord blood HSCs. After 20 days of co-culture, the expressions of the early marker of lymphopoiesis CD7 averaged 68.4% for HSC-Td20 and 70.8% for hPSCTd20 (Figure 4A). In addition, about 9% of the CD7^+^ cells expressed the CD4 and CD8 markers in HSC-Td20 versus 3.1% in hPSC-Td20. HSC or hPSC, induced in lymphopoiesis for 20 or 25 days, did not express CD3 and TCRαβ, indicating that the differentiated cells obtained were progenitors.

To further understand the identity of cells differentiated from hPSC or HSCs, we compared their transcriptomic signatures to natural thymocytes. For this, thymocytes were extracted from human thymuses and sorted on CD7 or CD4 and CD8 co-expression (Supplementary Figure S6). Hierarchical clustering defined four groups of cells: (1) D9 CD34^+^CD43^+^ EBs and Td15, (2) cord blood HSC and Td15, (3) HSC-Td15, HSC-Td25, Td20, and Td25, and (4) CD7^+^ and CD8^+^CD4^+^ thymocytes (Figure 4B). Pearson correlation and PCA analysis further confirmed a clear distinction between the EB D9 CD34^+^CD43^+^ and the HSC group. HSCs were closer to the iPSCTd15 cells. The iPSC-Td20, iPSC-Td25, HSC-Td15, and HSC-Td25 groups were closer to thymocytes (Figure 4B and Supplementary Figure S7). Notably, upon differentiation, the analysis highlighted the upregulation of specific pathways related to T cell biogenesis, such as “alpha-beta T cell differentiation, T cell receptor signaling, thymic T cell selection or MHC class I/II protein complex”, and the downregulation of pathways such as “hematopoietic stem cell differentiation or regulation of stem cell differentiation” (Figure 4C). Similar enrichment was found for HSC and hPSC differentiation. These results confirm the commitment of the cells to the T cell lineage.

**Figure 5:**
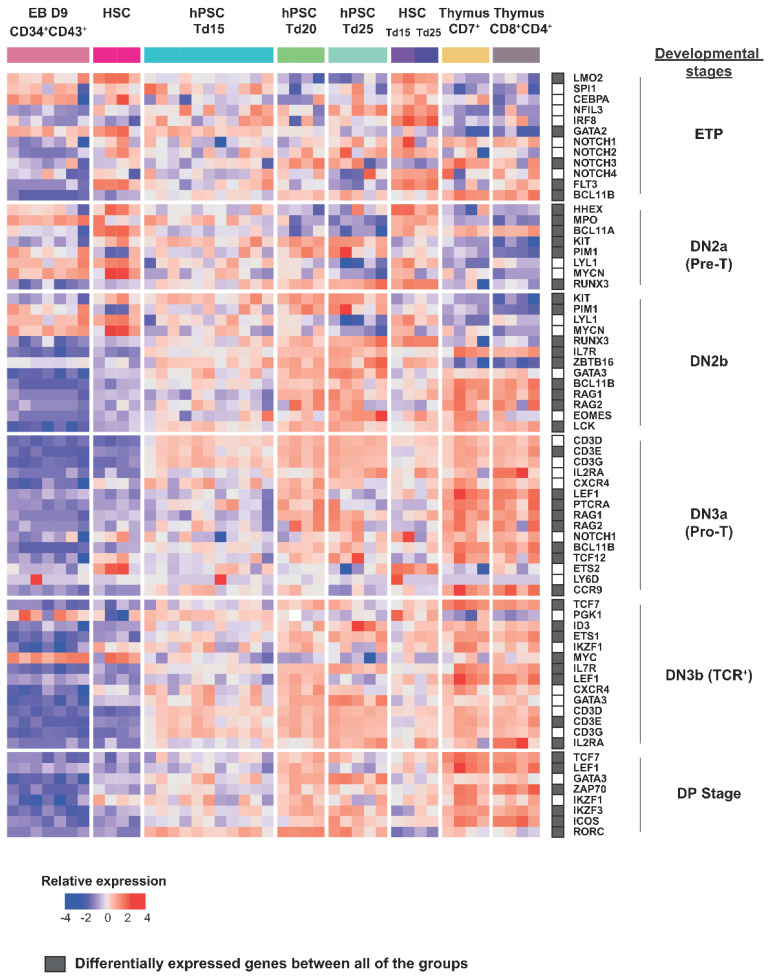
T-cell progenitors derived from hPSCs are stuck at the DN3a thymocyte stage. Gene expression heatmaps of selected markers associated with T cell development and function are shown as defined by DGE-seq. The heatmap was generated by scaling and center gene expression.

To further understand the developmental stage achieved by the in vitro differentiation of PSCs towards thymic lymphopoiesis, we then analyzed the stage-associated makers (Figure 5 and Supplementary Figure S8). T-cell progression through lymphopoiesis involves progressive phases of lineage specification, characterized by the acquisition of a T cell-specific transcriptional program. The different development stages are characterized as follows: the early intrathymic precursor of the ETP stage; the immediate pre-commitment DN2a stage (DN = double negative for mature T-cell coreceptors CD4 and CD8), also known as the pre-T stage; the newly committed DN2b stage; the DN3a stage that is the first major period of TCR gene rearrangement, also known as the pro-T stage; the DN3b and immature single-positive stages when cells proliferate vigorously following the first successful TCR locus gene rearrangement; and finally, the DP (CD4, CD8 double positive) stage when the second TCR locus is rearranged and cells successfully express their final TCR recognition specificity for the first time. The DP stage is the progenitor stage that gives rise to all effector subsets of T cells using the αβ form of the TCR [31]. We then analyzed genes well known to define the different developmental stages of T cells in the thymus [32,33,34]. Three profiles emerged from this analysis: (1) D9 CD34^+^CD43^+^ EBs and CD34^+^ cord blood HSCs, which overexpressed ETP and DN2a stage-related specific genes and downregulated other stage-related genes, (2) hPSC/HSC Td20, Td25, and thymic cells, which, on the contrary, downregulated ETP and DN2a stage-related genes and upregulated DN2b, DN3a, and DN3b stage-related genes, and (3) hPSCs-Td15, which have a particular intermediate profile (Figure 5). We noticed that the LMO2, GATA2, and MPO genes involved in the ETP and DN2a stages of differentiation [35,36,37] were upregulated in hiPSC-Td15, while they were downregulated in hiPSC-Td25 and HSC-Td25, as well as in CD7^+^ and CD8^+^CD4^+^ thymocytes. (Figure 5). On the other hand, genes such as IKZF3, CD3D, CD3E, RAG1, and RAG2 were significantly upregulated in hPSC-Td20 compared to hPSC-Td15 during differentiation. Similarly, BCL11B, GATA3 [38], IKZF3, and RAG1 were also significantly upregulated in hPSC-Td25 compared to hPSCTd15 and marked the transition to more advanced stages (Supplementary Figures S9 and S10). Moreover, we noticed that they also expressed the BCL11B and LCK genes, which are essential for the passage from DN2 to DN3. Interestingly, we observed that the hPSC/HSC Td20 and Td25 cells expressed RAG1/RAG2, as well as the three chains of CD3 (D/E/G), PTCRA, CD44, and MME, which are involved in the rearrangement of the TCR complex [39,40] (Figure 5 and Supplementary Figure S9). In addition, we could observe that differentiating cells, such as CD8^+^CD4^+^ thymocytes, expressed DN3b and DP stage-related genes such as TCF7, ZAP70, IKZF1 (IKAROS), IKZF3 (AIOLOS), or ICOS [41,42,43,44]. We found that there were no significant differences in the expressions of T-lineage genes such as BCL11B, LCK, ZAP70, and PTCRA between hPSC-Td25 and HSC-Td25 compared to CD8^+^CD4^+^ thymus, showing their engagement in the T lineage. (Supplementary Figures S9 and S10). We also saw that there were no significant differences in the expressions of these genes between hPSC-Td15 and HSC-Td15 and between hPSC-Td25 and HSC-Td25. T-hPSC-derived cell differentiation, therefore, behaved identically to HSC-derived cells. Since hPSC/HSC-Td20 and Td25 cells did not express the TCR/CD3 complex protein on the cell surface, the differentiation stage reached by hiPSC-Td25 and HSC-Td25 cells 249 was DN3a [45].

## 3. Discussion

In this study, we successfully and reproducibly generated T-cell progenitors at the DN3a stage from several hESC and hiPSC lines. While it is true that the method to induce mature T cells from TiPS cells has already been established by other groups, our analysis brings novel perspectives and is therefore quite different from that of other groups. Indeed, all our cultures up to T-cell differentiation were in feeder-free conditions. Moreover, we grew our hiPSCs on a Matrigel matrix, which greatly reduced the time of the cell culture and thus facilitated the launching of differentiations, which required a lot of time and cells. Two differentiation steps were necessary; the first one was the generation of HPSCs from hPSCs through embryonic body formation, and the second step was the differentiation of HPSCs into T cells through co-culture on OP9-DL1. The comparison of hPSCs-HPSCs with primary cord blood cells confirmed that hPSCs-HPSCs were similar to HSCs. Similarly, we confirmed that hPSC-Td25s were similar to primary thymocytes. Indeed, they recapitulated the transcriptome, protein markers, and differentiation potentials of primary cells. For this study, we generated T-iPSCs, which were similar to hESCs and other hiPSC lines.

We showed that T-iPSCs could differentiate similarly to hiPSCs reprogrammed from other cell types. Furthermore, it has been demonstrated that T-cell reprogramming can increase the lifespan of cells because newly produced cells have longer telomeres, which represents a significant therapeutic advantage [46]. To efficiently direct the differentiation of hiPSCs to the hematopoietic lineage, we used our optimized feeder-free protocol [30]. After 9 days of differentiation, we obtained CD34^+^CD45^+^ cells regardless of the hiPSCs or hESCs lines used. Discrepancies between HPSCs and CD34^+^ cord blood have been reported in particular on their multipotency and their ability to engraft in animals [47]. This can be explained by the fact that HPSCs differentiated from hPSCs recapitulate primitive rather than definitive hematopoiesis [48]. Therefore, our first endeavor was to assess HSPCs from iPSCs vs. cord blood. Functional analysis with a CFU assay showed similar potential: all CD34^+^ cells, regardless of the source, were able to multiply and differentiate in the whole repertoire of blood colonies. This suggests that the differentiated cells could be fully capable of differentiating into T cells.

The next step will be to produce cells with long-term renewal and engraftment potential in immunocompromised mice, without the challenging overexpression of numerous transgenes. Indeed, these protocols are still very laborious to perform. Moreover, the production of 11 kb lentivirus remains difficult, and 5 to 7 lentivirus are necessary [27,28,49]. The transcriptomic comparison between hiPSC-derived HPSCs with cord blood cells showed that CD34^+^CD43^+^ cells clustered with cord blood cells in contrast to CD34^+^CD43^−^ cells. Nevertheless, we observed that genes shown to be essential for grafting potential in animals by G. Daley’s team, such as the HOXA family and LCOR [27,28], were not expressed in CD34^+^CD43^+^. However, only one study so far has shown the generation of long-term multipotent HSPCs supporting hematopoietic reconstitution and self-renewal in vivo, but through early differentiated cells expressing APLNR, thus more likely an endothelial cell undergoing EHT or a newly formed HSPC [50]. D9 CD34^+^CD43^−^ EB cells had a hemogenic–endothelium profile and expressed APLRN, which may give them the potential to engraft in animals. It would be interesting to transplant D9 CD34^+^CD43^−^ EB cells in immunosuppressed animals and follow their differentiation by immunoprofiling and scRNA sequencing.

After establishing robust hematopoietic differentiation, we analyzed the induction of lymphopoiesis. After 20/25 days of co-culture on OP9-DLL1, we obtained CD7^+^, CD5^+^, CD4^+^, and CD8^+^ cells, which are major markers of T cells. We observed that hiPSC-Td20 upregulated CD8 over CD4, as previously reported [11,12,46,51,52]. Interestingly the upregulated pathways in hPSC-Td25 and HSC-Td25 were directed by genes involved in T-cell differentiation and in the establishment of the TCR and the MHC complex. Furthermore, we saw that hiPSC-Td25 and HSC-Td25 differentiated cells possessed the same abilities as cord blood HSCs to differentiate under our culture conditions. Even if the signature of the cells after 25 days of co-culture on OP9-DLL1 was clearly typical of thymic progenitors at the DN3a stage, our culture conditions did not allow at that stage the acquisition of a functional TCR either in HSC-TD25 or in T-iPSC-Td25. In addition, we observed, in our differentiation, cells expressing genes involved in ILC/NK development such as JCHAIN, RGPD3, TNFSF11, and KLRC1, which were significantly upregulated in hPSC-Td15/20/25 compared to CD7^+^ and CD8^+^CD4^+^ thymic controls (Supplementary Figures S8–S10) [11,30,46,52]. However, since we performed a bulk transcriptomic study, these expressions probably reflect a heterogeneous population rather than an aberrant cell type or commitment to another cell fate. We previously found that CD56^+^ cells represent around 25% of the CD7^+^CD8^+^ cell population [30]. One hypothesis could be that the CD56^+^ cells that we observed are part of a niche that limits T-cell development [46,52]. Moreover, studies have shown that a high expression of IL-7 could also prevent T cell selection [8,53,54,55]. Based on all of these results, we thus think the environment might be a critical limitation in T cell development in our setting and that it would be interesting to eliminate CD56^+^ cells from the culture, decrease the IL-7 concentration, and analyze the impact on TCR expression.

Advances in immune cell engineering are opening up new perspectives for the generation of custom synthetic T cells from hiPSCs. TCR acquisition during differentiation will no longer be an issue since antigenic specificity could be brought by other technical means, such as a transgenic TCR [24,56] or the use of chimeric antigen receptors (CARs) providing hiPSC-derived T cells with customizable antigen specificity in an HLA-independent manner. Indeed, Themeli et al. showed, in 2013, the possibility of inserting a CAR into hiPSCs under the control of an inducible promoter. The induction of the CAR during the differentiation resulted in the generation of functional CAR-T cells in vitro and in vivo efficient in a cancer model [52,57]. Today, most studies on T-cell differentiation from PSCs are conducted to generate cytotoxic effector T cells in the context of cancer immunotherapy. Indeed, PSCs represent an unlimited source of cells and allow for the generation of custom-modified T cells. Similarly, the use of Tregs derived from PSCs is also of great interest in the treatment of GVHD or autoimmune diseases with the same advantages [58]. It has been shown that the transduction of FOXP3 and transgenic TCR coding sequences into miPSCs, and then the differentiation of these cells, led to the generation of functional Tregs secreting regulatory molecules, such as IL-10 and TGF-β, and enabled the control of autoimmunity in an arthritic mouse model [59,60].

In conclusion, we generated a protocol for the differentiation of DN3a thymic progenitors from HSC, hESCs, and T-hiPSCs. The generation of T-cell progenitors in vitro offers the opportunity to better study and understand lymphopoiesis. Since it remains extremely difficult to study the genesis of T cells in humans, the in vitro generation of T cells would allow for the unprecedented investigation of lymphopoiesis to functionally test hypotheses. Altogether, our research represents an excellent model to study the establishment of TCR expression during T-cell differentiation in humans. This also opens new opportunities for drug screening and disease modeling and could provide an unlimited number of cells for regenerative medicine and cell therapy.

## 4. Materials and Methods

### 4.1. Cells

A total of 7 hiPSCs lines were used in this study, all of them derived from healthy donors. T04.01A, T04.01B, T05.003, and T05.006 came from human male adult T cells, MiPS209 and MiPS220 came from human female adult dermal fibroblasts [61], and LON71 came from human male adult dermal fibroblasts [62]. hES H9 cells (WA09 Lot WB0090) were obtained from the WiCell Research Institute, under authorization RE13-004 from the French embryo research oversight committee, Agence de la Biomédecine, and were also used in this study. Human somatic cells were obtained after the informed consent of patients.

OP9-DLL1/DLL4 stromal cells were kindly provided by Dr. Juan-Carlos Zunigo-Pflucker, University of Toronto.

Human thymus samples were obtained after childhood heart surgery at Nantes University Hospital (Pediatric cardiac surgery unit, CHU Nantes, Nantes, France).

### 4.2. Tissue Culture

hPSCs were cultured in feeder-free conditions on Matrigel (BD/Corning, Paris, France) in mTeSR1 (StemCell Technologies, Cambridge, UK); the cells were non-enzymatically dissociated with StemMACS passaging solution XF (Miltenyi Biotec, Paris, France) for passaging. OP9-DLL1/DLL4 cells were cultured in OP9 medium (α-MEM with 20% FBS, 2 mM l-glutamine) on gelatin-coated plates. All cells were cultured at 37 °C in normoxia (20% O_2_ and 5% CO_2_) and were tested for mycoplasma contamination regularly. Freshly extracted thymuses were collected in RPMI1640 medium supplemented with 10% FBS, 1 mML glutamine, and 0.5% penicillin–streptomycin. They were then shredded using needles to extract the cells before cell sorting by BD FACSAria (BD Biosciences, Paris, France).

### 4.3. Reprogramming of Human T Cells into T-iPSCs

Human T-iPSCs were established from T cells. In brief, hPBMCs were stimulated with coated antiCD3 and soluble anti-CD28 MAbs (1 µg/mL each) in complete T-cell medium (RPMI1640 medium, 10% AB serum, 1% nonessential amino acids, 1% sodium pyruvate, 1% hepes, 2 mM L-glutamine, and 1% penicillin–streptomycin) supplemented with IL-2 (1000 U/mL). After four days of stimulation, 98% CD3^+^TCRαβ^+^ cells were obtained from the culture. Then, 10^5^ activated T cells were seeded per well on a 96-well flat-bottom plate in complete RPMI1640 medium with 10% AB serum and IL-2 (1000 U/mL) with coated anti-CD3 and soluble anti-CD28 MAbs (1 µg/mL each) and transduced with three Sendai virus vectors encoding for polycistronic Klf4-Oct4-Sox2, cMyc, and Klf4 at MOIs of 6-6 and 3.6, respectively. On day 3 of transduction, the cells were seeded on 35 mm dishes coated with irradiated mouse feeder cells and cultured in T-cell medium. The medium was changed to hPSC medium (DMEM/F12 supplemented with 20% knockout serum replacer, 2 mM L-glutamine, 1% nonessential amino acids, 10 μM 2-mercaptoethanol, and 5 ng/mL basic fibroblast growth factor (FGF2)) on day 5 and refreshed daily. T-iPSC colonies appeared at ~13–25 days after transduction. All T-iPSC lines were mechanically passaged to fresh feeder-coated tissue culture dishes with hPSC medium every 6–7 days. After about 10 passages, colonies were picked on MEF and put directly on Matrigel-coated dishes in TeSR1 medium. We waited about 5–6 passes before use for differentiation.

### 4.4. Early Germ Layer Differentiation

hPSC lines were differentiated into endoderm, mesoderm, and ectoderm using a Stemmacs Trilineage Kit (Miltenyi Biotec, Paris, France). About 80,000 cells for the mesoderm, 130,000 cells for the endoderm, and 100,000 cells for the ectoderm were plated in 24-well plates, and cultured in specific media for 7 days, as specified by the protocol. On day 7, differentiated cells were lysed and analyzed by ARN-DGE sequencing.

### 4.5. SNP Analysis

DNA was extracted from somatic and iPSCs samples using the QIAGEN QiaAmp kit, according to the manufacturer’s recommendations. The gDNA was quantified and qualified using a nanodrop. Then, 200 ng of gDNA was outsourced (Integragen, Evry, France) for karyotype analysis using HumanCore-24-v1 SNP arrays. This array contains over 300,000 probes distributed throughout the genome, with a median coverage of one probe every 5700 bases. All genomic positions were based on Human Genome Build 37 (hg19). The DNA samples were hybridized on HumanCore-24-V1 SNP arrays according to the manufacturer’s instructions by Integragen. Analysis was performed with GenomeStudio software (Illumina, San Diego, USA). Chromosome abnormalities were determined by visual inspection of the logR ratios and B-allele frequency (BAF) values and comparing parental cells and iPS-derived samples. The logR ratio, the ratio between the observed and expected probe intensity, is informative regarding copy number variation (i.e., deletions/duplications), and BAF is informative regarding heterozygosity. We used the SNP data to compute CNV. In particular, this type of chip allows for detecting the loss of heterozygosity (LOH), an important concern for hiPSCs, which is not possible with classical CGH arrays.

### 4.6. Hematopoietic T-Cell Differentiation from PSCs

For the differentiation of hPSCs to hematopoietic precursors, we used an optimized serum- and feeder-free in vitro differentiation protocol [30]. Briefly, undifferentiated hPSC colonies were treated with StemMacs passaging solution XF (Miltenyi Biotec, Paris, France) for 3 min, rinsed with α-MEM (Life Technologies), and transferred to low-attachment plates to allow for the formation of embryoid bodies (EBs) in mTeSR1 (StemCell Technologies, Cambridge, UK). The formation of EBs was facilitated by 18 h of incubation in the presence of 10µM of Y-27632 dihydrochloride (Axon Medchem, Groningen, Netherlands). On day 0, the embryoid bodies were cultured in EB medium (StemPro34, Life Technologies, with 2 mM l-glutamine, 1% nonessential amino acids, 50 μM 2mercaptoethanol, 1% penicillin–streptomycin, and 50 μg/mL ascorbic acid) with 30 ng/mL of hBMP4. On day 1, the EBs were then cultured with hBMP-4 and 5 ng/mL of hFGF2 until day 3 to allow for mesoderm induction. Next, hematopoietic specification and expansion were achieved in the presence of hVEGF (20 ng/mL) and a cocktail of hematopoietic cytokines (hSCF 100 ng/mL, hFlt3L 20 ng/mL, hIL-3 20 ng/mL, and hFGF2 5 ng/mL) until day 7, and after, continued without hFGF2.

On day 9, the EBs containing hematopoietic progenitor cells were dissociated with treatment with Accutase for 15 min, and single cells were then seeded on OP9-DLL1/DLL4 monolayers to allow for their T lymphoid differentiation in OP9 medium (α-MEM with 20% FBS, 2 mM l-glutamine, 1% nonessential amino acids, 50 μM 2-mercaptoethanol, 1% penicillin–streptomycin, and 50 μg/mL ascorbic acid) supplemented with hSCF 10 ng/mL, hIL-7 5 ng/mL, and hFlt3L 10 ng/mL. All recombinant factors were purchased from PeproTech (Peprotech, Paris, France), with the exception of hBMP-4 from R&D Systems (Abingdon, UK).

### 4.7. CFU Assay

MethoCult colony-forming unit (CFU) assays were performed with MethoCult SF H4636 (StemCell Technologies, Cambridge, UK) according to the manufacturer’s protocol. Briefly, on day 9, cells from EBs were dissociated with Accutase and CD34^+^ cells were MACS-purified with the human CD34 UltraPure MicroBead Kit (Miltenyi Biotech, Paris, France). After washing, 10,000 cells were resuspended in 300 mL EB medium, gently mixed with 3 mL MethoCult, and plated onto 2.35 mm dishes. Cord blood CD34^+^ cells (2C-101, Lonza, Colmar, France) were thawed according to the manufacturer’s instructions, washed in DPBS, and 2000 cells were seeded as described above. The cells were incubated at 37 °C and 5% CO_2_ for 14 days. The total colony numbers were quantified in duplicate plates using an inverted light microscope, and the average number of CFUs per plate was determined. The MethoCult was then rehydrated to recover the cells. The cells were washed 3x with DPBS and then analyzed by flow cytometry for the expressions of CD15, CD14, and CD235a on the different cell types.

### 4.8. FACS Analysis

The following conjugated antibodies were used for flow cytometric phenotyping and analysis: anti-CD34, anti-CD43, anti-KDR, anti-CD45, anti-CD7, anti-CD5, anti-CD4, anti-CD8, anti-CD3, antiTCRαβ, anti-CD56, anti-CD15, anti-CD14, and anti-CD235a purchased from BD Biosciences. All antibodies were used in a 1:30 dilution. Dead cells were excluded from analysis in all experiments by staining with DAPI. Flow cytometry analysis was conducted on an LSRII cytometer (BD Biosciences, Paris, France), a FACS CANTO (BD Biosciences, Paris, France), or a FACS CELESTA (BD Biosciences, Paris, France) and analyzed using FlowJo software (BD Biosciences, Ashland, OR, USA).

### 4.9. Extraction of RNA and DNA

The total RNA and DNA were extracted using an RNeasy^®^-free DNase kit (Qiagen, Hilden, Germany) and a QIAamp DNA mini kit (Qiagen, Hilden, Germany), respectively.

### 4.10. 3′ Digital Gene Expression (3′ DGE) RNA Sequencing

Total RNA molecules were extracted from the cells with RNeasy-Mini kits (Qiagen). The protocol of 3′ DGE RNA sequencing was performed as previously described in [61]. The libraries were then sequenced on a NovaSeq 6000 (Illumina, San Diego, USA). Reading frame 1 encodes for specific barcodes and unique molecular identifiers (UMIs) and reading frame 2 encodes for the 3′RNA region. The data were aligned along the human genome reference (hg19) and a count matrix was generated by counting the sample-specific UMIs associated with the genes for each sample. Samples with fewer than 200,000 UMIs and fewer than 5000 genes expressed were excluded from the analysis. Then, a batch correction between the samples of different experiments was applied. A principal component analysis (PCA) was performed in order to visualize the samples’ repartition by reducing the number of dimensions. Correlations between the samples were assessed with Pearson’s linear correlation heatmaps. Higher correlations are marked in yellow and lower correlations are in red. Differentially expressed genes between conditions were calculated using R package Deseq2 (v4.0, Bioconductor, Buffalo, USA) by first applying a variance-stabilizing transformation (vst). Genes with an adjusted *p*-value inferior to 0.05 were considered differentially expressed genes. Gene expressions were visualized with heatmaps that were generated by scaling and center gene expression. Finally, pathways analysis was performed: R package “Fgsea” and databases such as Kegg, Reactome, and Gene Ontology were used to identify significantly enriched or depleted groups of genes in each condition. The accession number for DGE-RNA sequencing raw data is n° PRJEB46691.

### 4.11. High-Throughput Sequencing of the TCRβ Chain

Genomic DNA was extracted from cells with a QIAamp DNA mini kit (QIAGEN). The ImmunoSEQ hsTCRB kit (Adaptive Biotechnologies, Seattle, Washington, DC, USA) was used according to the manufacturer’s protocol. A DNA quantity of 21 ng per sample was defined, and four genomic replicates per sample were separately amplified in a two-step multiplex PCR, adding adaptor sequences containing sample-identifying barcodes. Then, the library was sequenced on a Miseq (150 cycles v3, Injection of 18 pM + 5% PhIX, sequencing pair-end 156-00-12). Raw sequencing run folders were subsequently uploaded to Adaptive Biotechnologies and processed through their in-house pipeline. The results were then further processed via Adaptive Biotechnologies Analyzer (v3.0, Adaptive Biotechnologies, Seattle, Washington, DC, USA).

## Data Availability

Data will be available upon reasonable request to the corresponding authors.

## Supplementary Materials

The following supporting information can be downloaded at: www.mdpi.com/article/10.3390/cells11244046/s1. Figure S1: Expressions of CD34 and CD43 in HPSCs differentiated from several hiPSC sources; Figure S2: Multipotent HPSCs derived from hPSCs; Figure S3: Gating strategy of multipotent HPSCs derived from hPSC sorting; Figure S4: Principal component analysis during HPSC differentiation; Figure S5: Molecular characterization of HSPCs derived from hPSCs; Figure S6: Gating strategy of thymocytes sorting; Figure S7: Principal component analysis during T cell differentiation; Figure S8: T-cell progenitor derived from hPSCs are stuck at the DN3a thymocyte stage; Figure S9: Molecular characterization of T-cell progenitors derived from hPSCs; Figure S10: Summary of statistical significance of difference for each sample in T-cell differentiation.
