# Supplementary material for: Generation of CD34+CD43+ Hematopoietic Progenitors to Induce Thymocytes from Human Pluripotent Stem Cells
Source: Cells. 2022 Dec 14;11(24):4046. doi: 10.3390/cells11244046 (PMC9777438; doi:10.3390/cells11244046)
Supplement: Supplementary file 1 [file cells-11-04046-s001.zip › cells-2010483-supplementary.pdf]

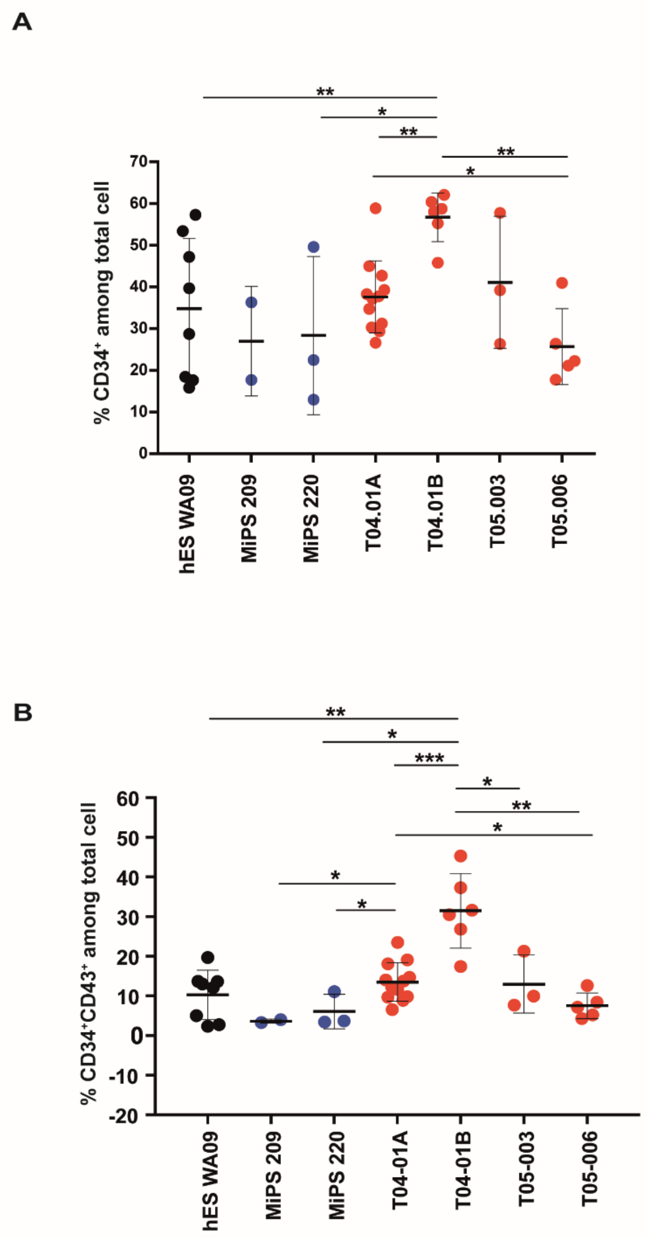

Supplementary Figure S1: Expression of CD34 and CD43 in HPSCs differentiated from several hiPSCs sources

A, Percent of total cells from day 9 EBs expressing CD34, mean +/- SEM are represented. Mann Whitney test, *p<0,05, **p<0,001, ***p<0, 0001. **B**, Percent of total cells from day 9 EBs

expressing CD34 and CD43, mean +/- SEM are represented. Mann Whitney test, *p<0,05, **p<0,001, ***p<0, 0001.

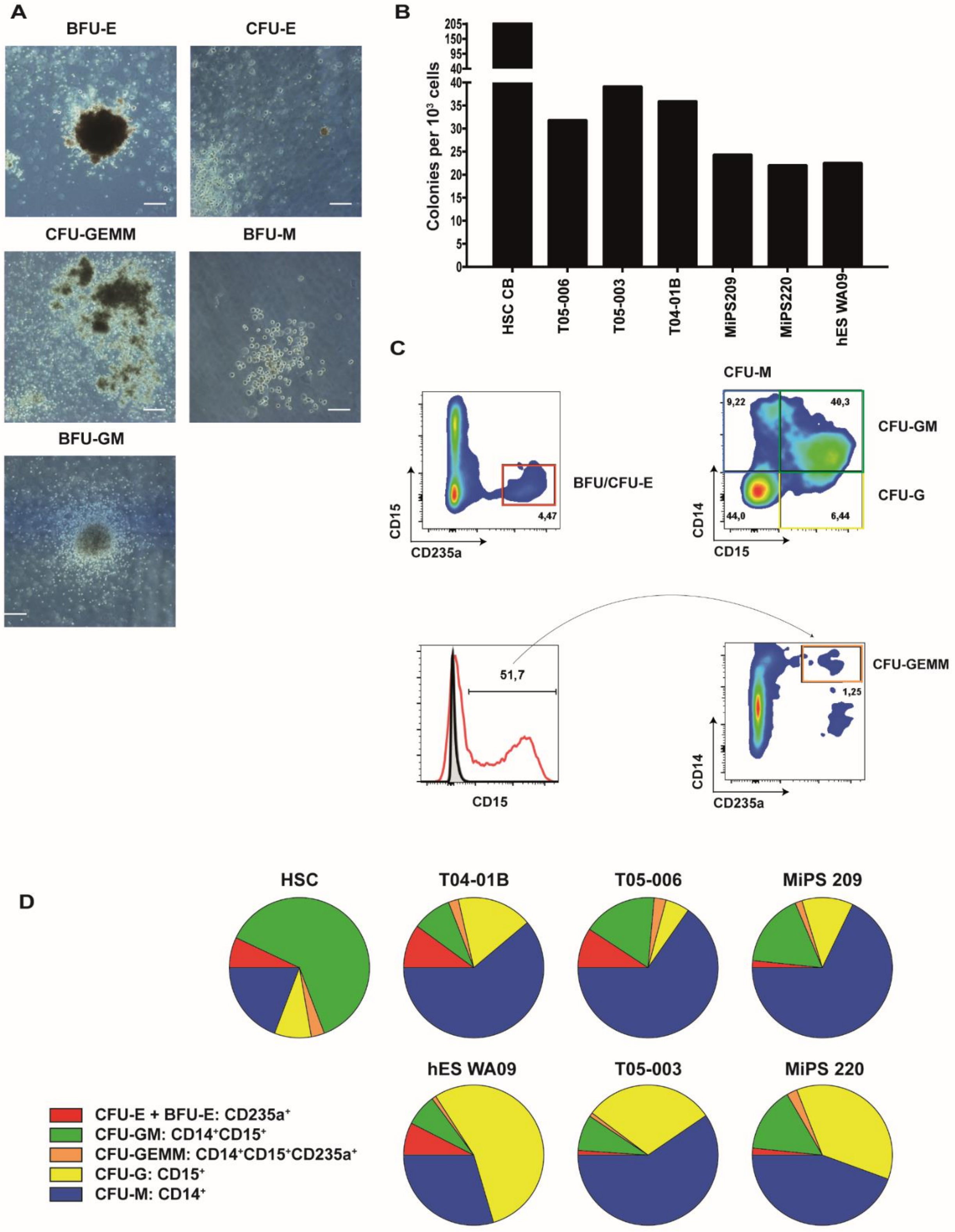
A
BFU-E
CFU-E
CFU-GEMM
BFU-M
BFU-GM
B
Colonies per 10³ cells
HSC CB
T05-006
T05-003
T04-01B
MiPS209
MiPS220
hES WA09
C
BFU/CFU-E
CD15
CD235a
4,47
CFU-M
9,22
40,3
CFU-GM
CFU-G
44,0
6,44
CD14
CD15
51,7
CD15
CFU-GEMM
1,25
CD14
CD235a
D
HSC
T04-01B
T05-006
MiPS 209
hES WA09
T05-003
MiPS 220
CFU-E + BFU-E: CD235a⁺
CFU-GM: CD14⁺CD15⁺
CFU-GEMM: CD14⁺CD15⁺CD235a⁺
CFU-G: CD15⁺
CFU-M: CD14⁺

Supplementary Figure S2: Multipotent HPSCs derived from hPSCs

A, Representative photos of blood colonies BFU-E, CFU-E, CFU-GEMM, CFU-M and CFU- GM from hPSCs. B, Total colonies number per 1000 CD34+ cells seeded cells in methyl-cellulose in indicated cell lines. C, After 14 days in methyl-cellulose, the cells are analyzed by flow-cytometry for the expression of CD14, CD15 and CD235a markers and according to the following gating strategy. The combination of these markers allows to determine the different blood colonies. D, Summary of flow-cytometry analysis of CFU assay blood colonies for the indicated cell lines.

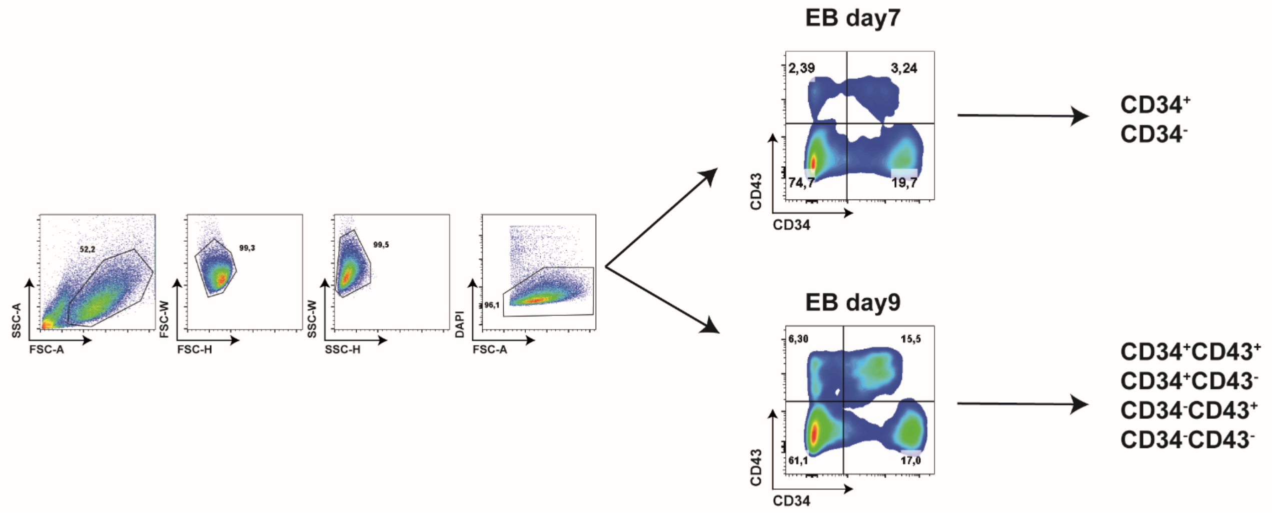

Supplementary Figure S3: Gating strategy of multipotent HPSCs derived from hPSCs sorting

Gating strategy for cell sorting by FACS Aria. Cells were selected on morphology, exclusion of doublets and dead cells (DAPI), and expression of CD34 et CD43.

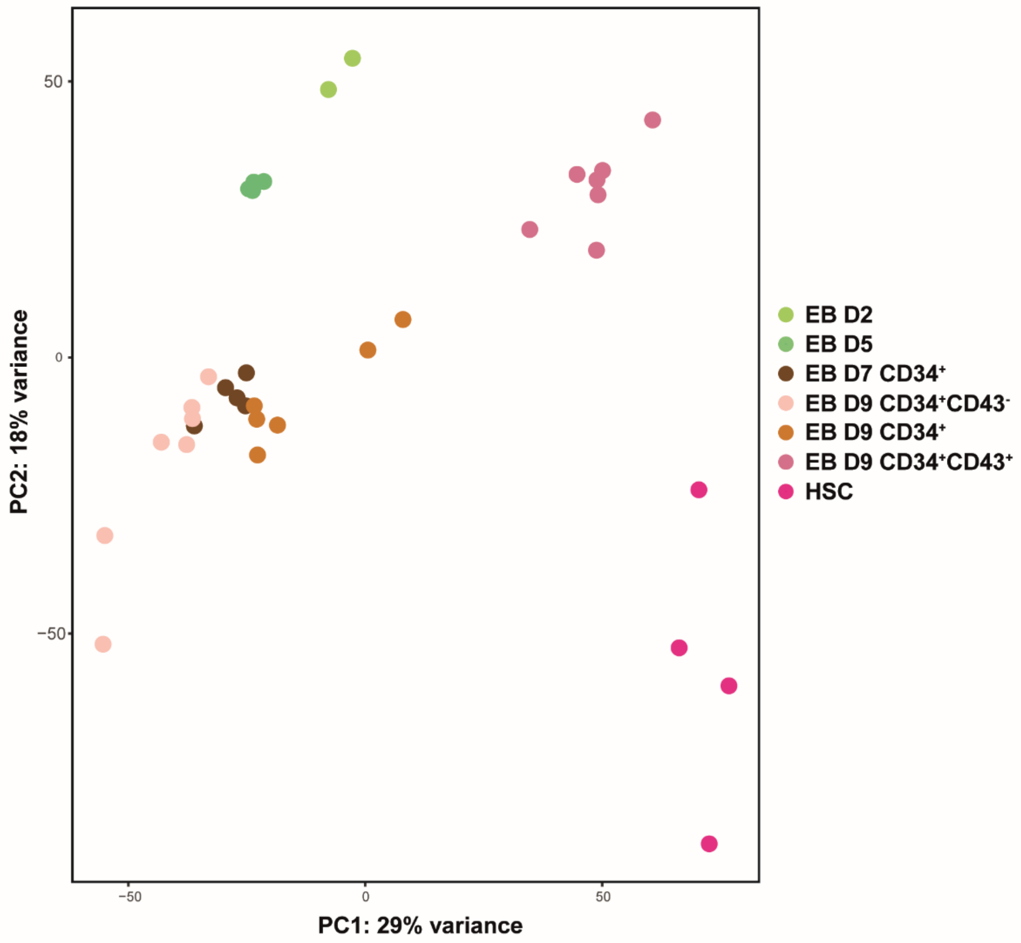

Supplementary Figure S4: Principal component analysis during HPSCs differentiation

PCA analysis of EB D2 (n=2), EB D5 (n=4), EB D7 CD34+ (n=5), EB D9 CD34+CD43- (n=7), EB D9 CD34+ (n=6), EB D9 CD34+CD43+ (n=7) and HSC (n=4) during differentiation. PC1and PC2 are displayed for differentiated cells. PC1 accounted for 29% of the variance and explained the stages of differentiation. PC2 accounted for 18% variance and segregated the primary cells from the differentiated cells.

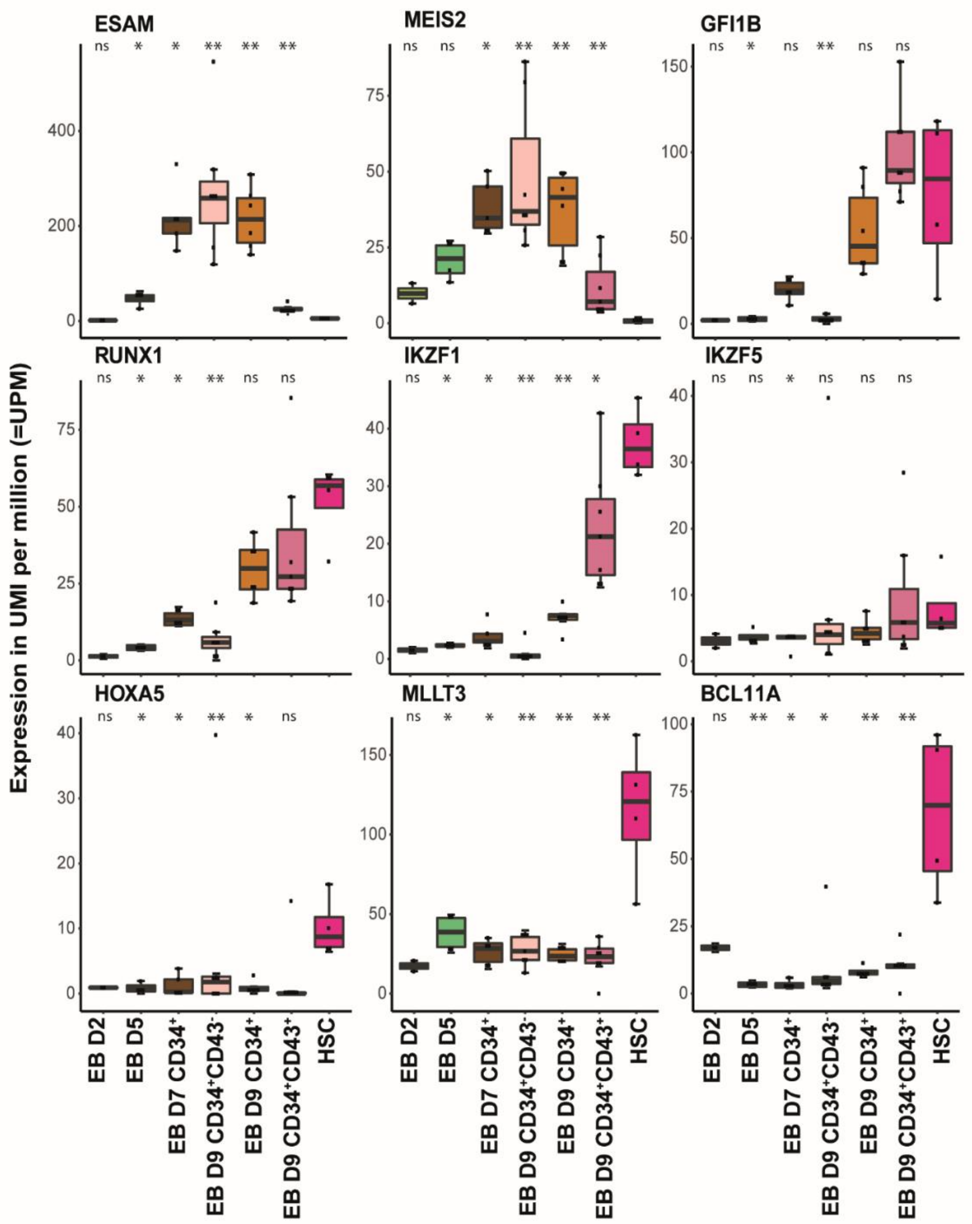

Supplementary Figure S5: Molecular characterization of HSPCs derived form hPSCs

Gene expression levels of indicated lineage markers are shown for EB D2 (n=2), EB D5 (n=4), EB D7 CD34+ (n=5), EB D9 CD34+CD43- (n=7), EB D9 CD34+ (n=6) and EB D9 CD34+CD43+ (n=7) samples and HSC (n=4) are included as control. Expression levels are given as number of

transcripts per million of mRNA molecules. In each boxplot, the top and bottom of the box represent the third and first quartile, respectively; the band represents the median; and error bars show the interquartile range. A Wilcoxon-Mann-Whitney statistical test was performed for each sample, with HSC taken as the reference group. Asterisks indicate statistical significance of the difference: *p value < 0.05, **p value < 0.01, ***p value < 0.001.

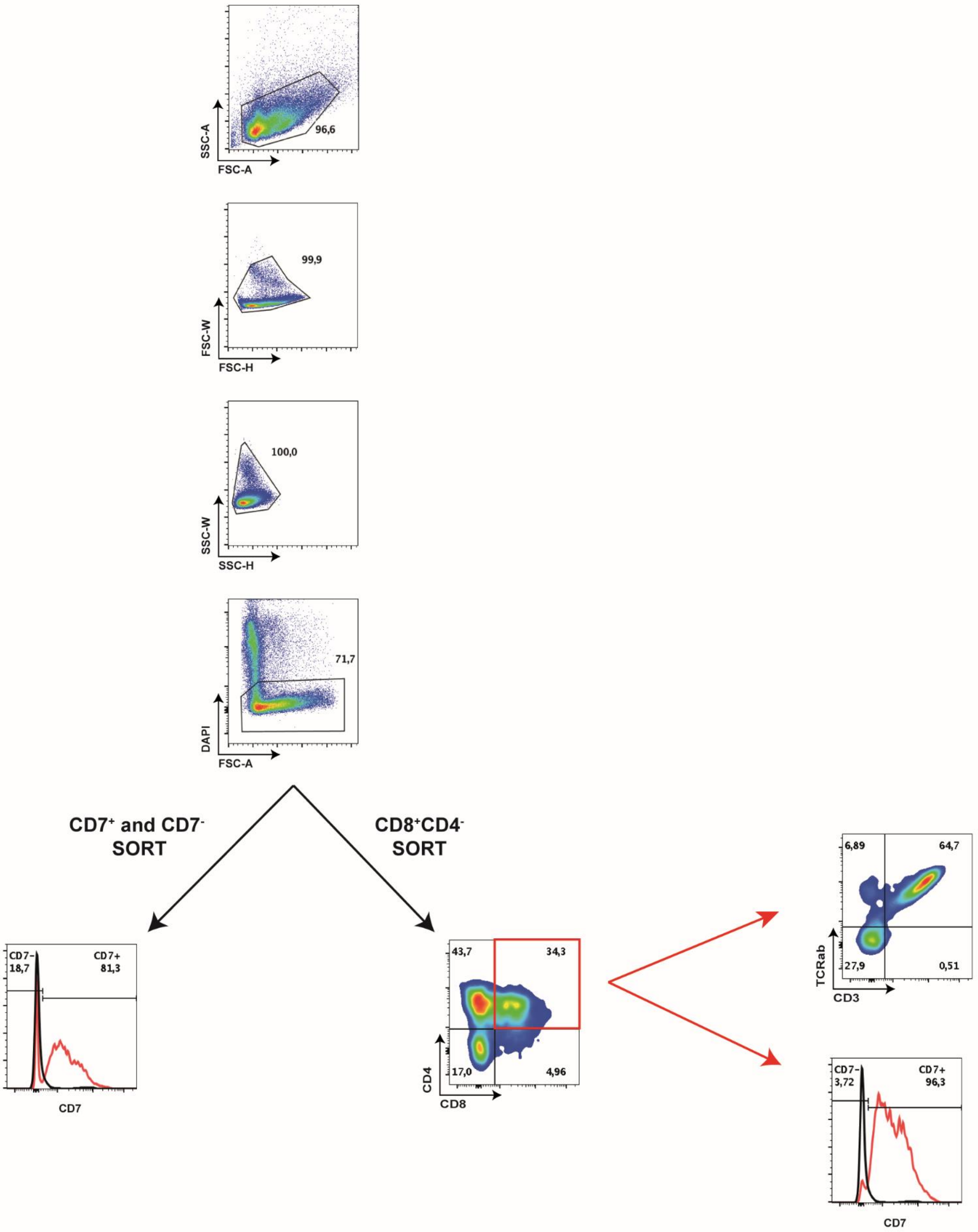
SSC-A
FSC-A
96,6
FSC-W
FSC-H
99,9
SSC-W
SSC-H
100,0
DAPI
FSC-A
71,7
CD7⁺ and CD7⁻ SORT
CD8⁺CD4⁻ SORT
CD7−
18,7
CD7+
81,3
CD7
43,7
34,3
17,0
4,96
CD4
CD8
6,89
64,7
27,9
0,51
TCRab
CD3
CD7−
3,72
CD7+
96,3
CD7

Supplementary Figure S6: Gating strategy of thymocytes sorting

Gating strategy for cell sorting by FACS Aria. Cells were selected on morphology, exclusion of doublets and dead cells (DAPI), and expression of CD7 or CD8 et CD4. 96,3% of $CD8^{+}CD4^{+}$ expressed CD7 and 64,7% CD3 and TCRαβ.

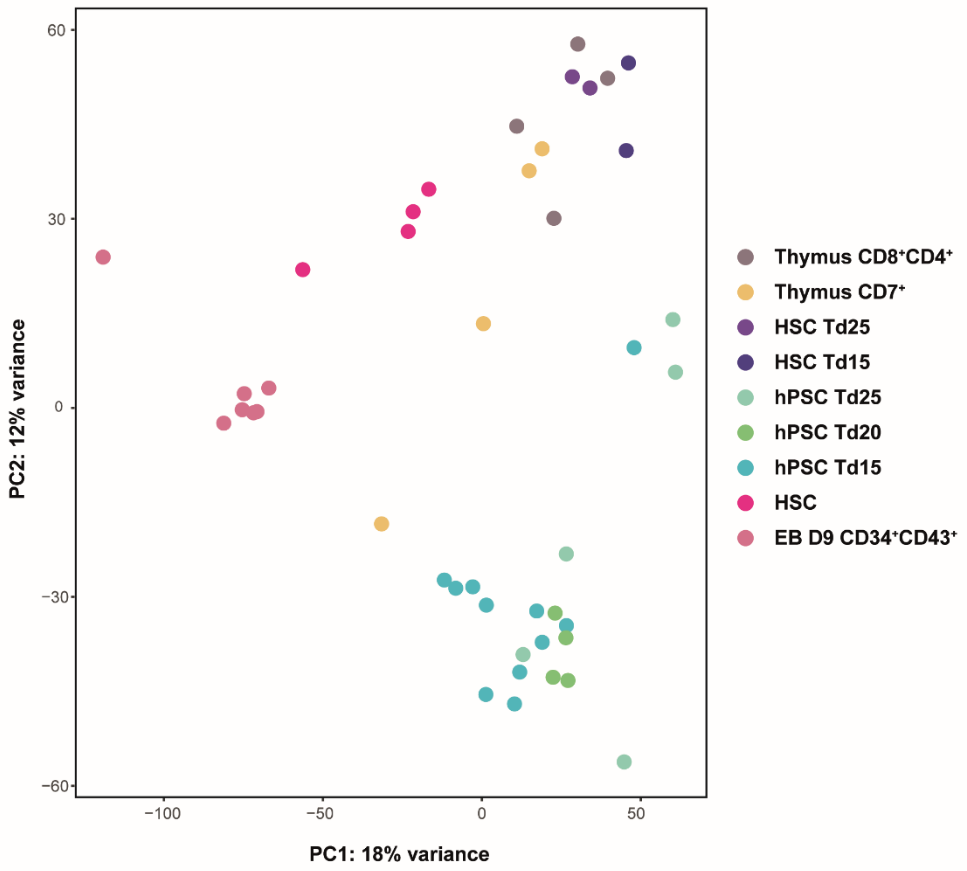

Supplementary Figure S7: Principal component analysis during T cells differentiation

PCA analysis of EB D9 CD34+CD43+ (n=7), HSC n=4, hPSC Td15 (n=11), hPSC Td20 (n=4), hPSC Td25 (n=5), HSC Td15 (n=2), HSC Td25 (n=2), thymocytes CD7+ (n=4) and thymocytes CD8+CD4+ (n=4) during differentiation. PC1and PC2 are displayed for differentiated cells. PC1 accounted for 18% of the variance and explained the stages of differentiation. PC2 accounted for 12% variance and segregated the primary cells from the differentiated cells.

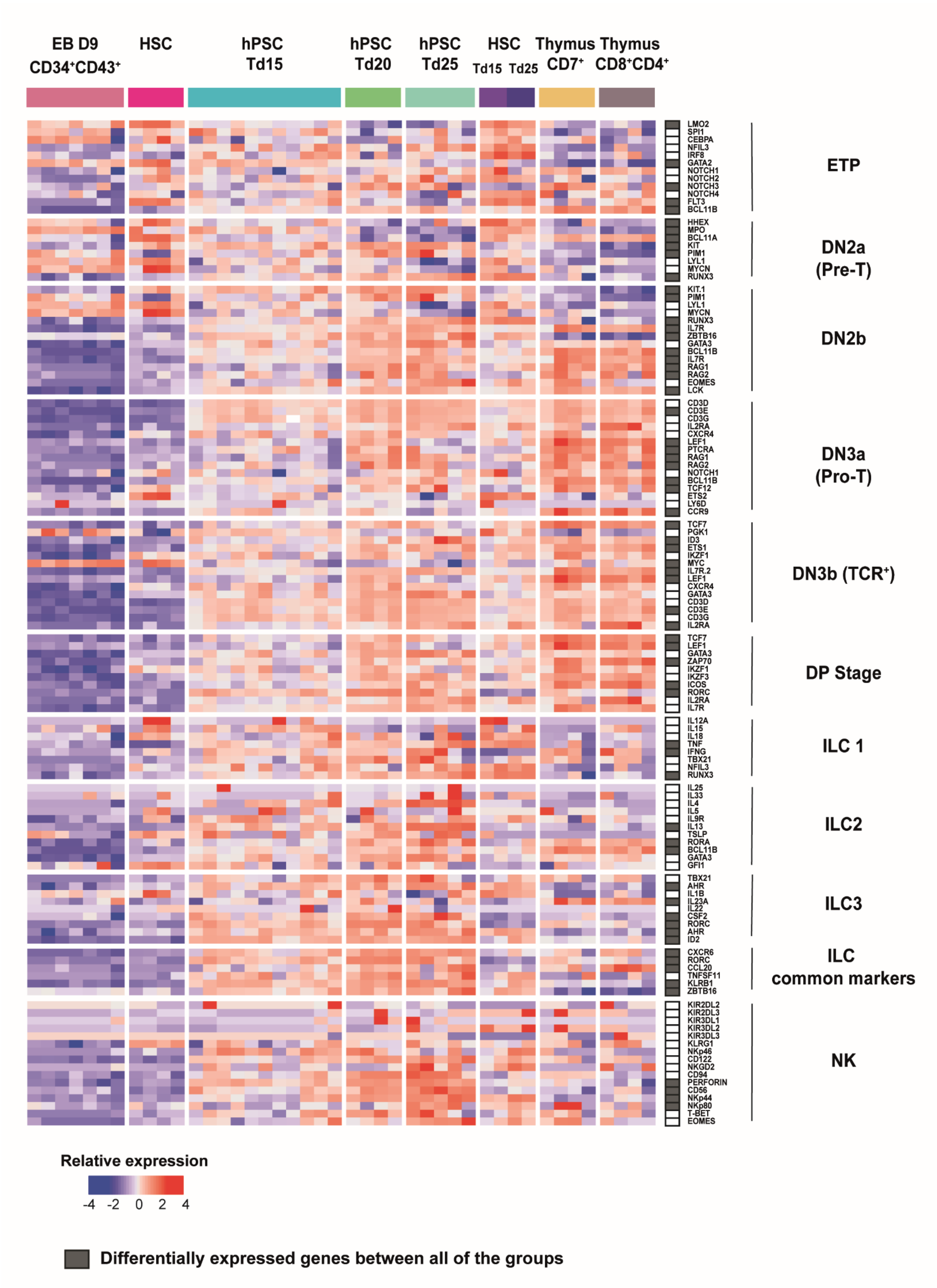

EB D9 CD34+CD43+
HSC
hPSC Td15
hPSC Td20
hPSC Td25
HSC Td15 Td25
Thymus CD7+
Thymus CD8+CD4+
LMO2
SPI1
CEBPA
NFIL3
IRF8
GATA2
NOTCH1
NOTCH2
NOTCH3
NOTCH4
FLT3
BCL11B
ETP
HHEX
MPO
BCL11A
KIT
PIM1
LYL1
MYCN
RUNX3
DN2a (Pre-T)
KIT.1
PIM1
LYL1
MYCN
RUNX3
IL7R
ZBTB16
GATA3
BCL11B
IL7R
RAG1
RAG2
EOMES
LCK
DN2b
CD3D
CD3E
CD3G
IL2RA
CXCR4
LEF1
PTCRA
RAG1
RAG2
NOTCH1
BCL11B
TCF12
ETS2
LY6D
CCR9
DN3a (Pro-T)
TCF7
PGK1
ID3
ETS1
IKZF1
MYC
IL7R.2
LEF1
CXCR4
GATA3
CD3D
CD3E
CD3G
IL2RA
DN3b (TCR+)
TCF7
LEF1
GATA3
ZAP70
IKZF1
IKZF3
ICOS
RORC
IL2RA
IL7R
DP Stage
IL12A
IL15
IL18
TNF
IFNG
TBX21
NFIL3
RUNX3
ILC 1
IL25
IL33
IL4
IL5
IL9R
IL13
TSLP
RORA
BCL11B
GATA3
GFI1
ILC2
TBX21
AHR
IL1B
IL23A
IL22
CSF2
RORC
AHR
ID2
ILC3
CXCR6
RORC
CCL20
TNFSF11
KLRB1
ZBTB16
ILC common markers
KIR2DL2
KIR2DL3
KIR3DL1
KIR3DL2
KIR3DL3
KLRG1
NKp46
CD122
NKGD2
CD94
PERFORIN
CD56
NKp44
NKp80
T-BET
EOMES
NK
Relative expression
-4 -2 0 2 4
Differentially expressed genes between all of the groups

Supplementary Figure S8: T-cell progenitor derived from hPSC are stuck at the DN3a thymocyte stage

Gene expression heatmaps of selected markers associated in T-cells, Innate cells like (ILCs) and Natural Killer cells (NKs) development and function are shown defined by DGE-seq.

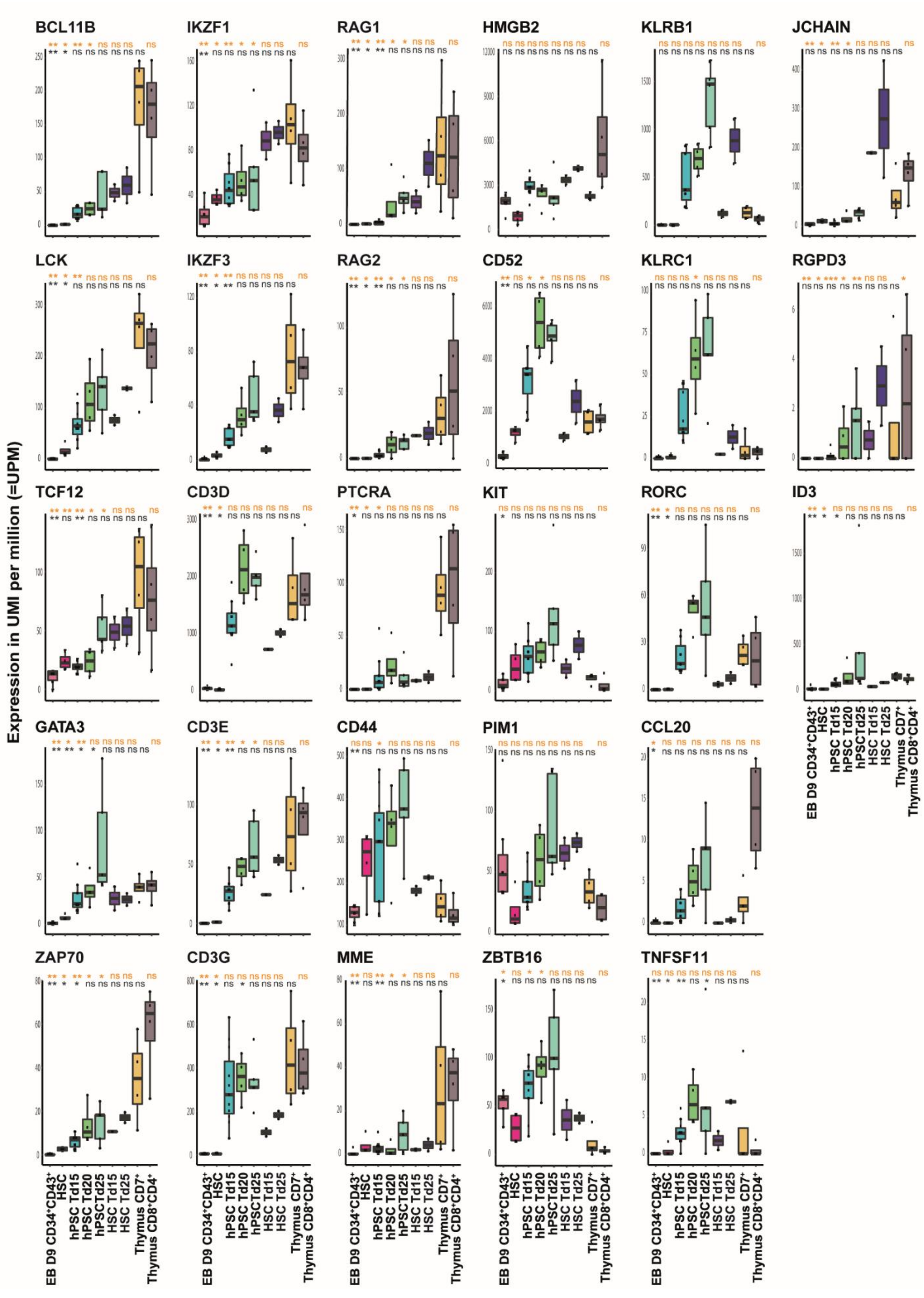
BCL11B
IKZF1
RAG1
HMGB2
KLRB1
JCHAIN
LCK
IKZF3
RAG2
CD52
KLRC1
RGPD3
TCF12
CD3D
PTCRA
KIT
RORC
ID3
GATA3
CD3E
CD44
PIM1
CCL20
ZAP70
CD3G
MME
ZBTB16
TNFSF11
Expression in UMI per million (=UPM)
EB D9 CD34+CD43+
HSC
hPSC Td15
hPSC Td20
hPSCTd25
HSC Td15
HSC Td25
Thymus CD7+
Thymus CD8+CD4+

Supplementary Figure S9: Molecular characterization of T-cell progenitors derived form hPSCs

Gene expression levels of indicated lineage markers are shown for EB D9 CD34+CD43+ (n=7), HSC n=4, hPSC Td15 (n=11), hPSC Td20 (n=4), hPSC Td25 (n=5), HSC Td15 (n=2), HSC Td25 (n=2) and thymocytes CD7+ (n=4) and CD8+CD4+ (n=4) are included as control. Expression levels are given as number of transcripts per million of mRNA molecules. In each boxplot, the top and bottom of the box represent the third and first quartile, respectively; the band represents the median; and error bars show the interquartile range. A Wilcoxon-Mann-Whitney statistical test was performed for each sample, with thymocytes CD7+ (orange) and CD8+CD4+ (grey) taken as the reference group. Asterisks indicate statistical significance of the difference: *p value < 0.05, **p value < 0.01, ***p value < 0.001.

			BCLL1B	LCK	TCF12	GATA3	ZAP70	IKZF1	IKZF3	CD3D	CD3E	CD3G	RAG1	RAG2	PTCRA	CD44	MME	HMGB2	CD52	KIT	PIM1	ZBTB16	KLRB1	KLRC1	RORC	CCL20	TNFSF11	JCHAIN	RGPD3	ID3
EB CD34+CD43+	vs	HSC	**	**	**	**	**	*	*		**		**				*	*	**		*	*		*						
EB CD34+CD43+	vs	hPSC-Td15	****	****	**	****	****	**	****	****	****	****	**	**	**	**	*	**	****	***	*	*	****	****	****	**	***			
EB CD34+CD43+	vs	hPSC-Td20	**	**		**	**	*	**	**	**	**	**	**	**	**			**	**			**	**	**	**	**			
EB CD34+CD43+	vs	hPSC-Td25	**	**	**		**	*	**	**	**	**	**	*	**				**					**		*				
EB CD34+CD43+	vs	HSC-Td15	*	*			*				*		*	*	*															
EB CD34+CD43+	vs	HSC-Td25	*	*			*				*		*	*	*									*						
EB CD34+CD43+	vs	Thymus CD7+	**	**	**	**	**	**	**	**	**	**	**	**	**		**		**			*			**	*		**	**	**
EB CD34+CD43+	vs	Thymus CD8+CD4+	**	**	**	**	**	**	**	**	**	**	**	**	*	**	**		**	*					**	*	**			**
HSC	vs	hPSC-Td15	**	**		**			**	**	**	**			*			**	**			*	**	**	**	*	*			
HSC	vs	hPSC-Td20	*	*		*	*		*	*	*	*	*	*	*				*		*	*	*	*	*	*	*			
HSC	vs	hPSC-Td25	*	*					*	*	*	*	*		*				*					*		*				
HSC	vs	HSC-Td15																												
HSC	vs	HSC-Td25																												
HSC	vs	Thymus CD7+	*	*	**	*	*	*	*	*	*	*	*		*										*			*	*	*
HSC	vs	Thymus CD8+CD4+	*	*		**	*		*	*	*	*	*	*											*		*			*
hPSC-Td15	vs	hPSC-Td20							**	**	**		**	**					**		*			*	*		**			
hPSC-Td15	vs	hPSC-Td25	**		*				***				***																	
hPSC-Td15	vs	HSC-Td15	*																											
hPSC-Td15	vs	HSC-Td25	**		*			*					*	*							*									
hPSC-Td15	vs	Thymus CD7+	**	**	**		**	**	**		**		**	**	*	*	**		*			**		**				**	***	
hPSC-Td15	vs	Thymus CD8+CD4+			**	*	*		**		**		*	**			*										**			*
hPSC-Td20	vs	hPSC-Td25																												
hPSC-Td20	vs	HSC-Td15																												
hPSC-Td20	vs	HSC-Td25																												
hPSC-Td20	vs	Thymus CD7+	*		*			*			*		*	*			*		*			*		*				*	*	
hPSC-Td20	vs	Thymus CD8+CD4+										*																		
hPSC-Td25	vs	HSC-Td15																												
hPSC-Td25	vs	HSC-Td25																												
hPSC-Td25	vs	Thymus CD7+			*			*			*		*	*			*												**	
hPSC-Td25	vs	Thymus CD8+CD4+																									*			
HSC-Td15	vs	HSC-Td25																												
HSC-Td15	vs	Thymus CD7+																												
HSC-Td15	vs	Thymus CD8+CD4+																												
HSC-Td25	vs	Thymus CD7+																												
HSC-Td25	vs	Thymus CD8+CD4+																												
Thymus CD7+	vs	Thymus CD8+CD4+																											*	

Supplementary Figure S10 (related to supplementary figure 9): Summary of Statistical Significance of Difference for each sample in T-cell differentiation

Gene expression levels of indicated lineage markers are shown for EB D9 CD34+CD43+ (n=7), HSC n=4, hPSC Td15 (n=11), hPSC Td20 (n=4), hPSC Td25 (n=5), HSC Td15 (n=2), HSC Td25 (n=2) and thymocytes CD7+ (n=4) and CD8+CD4+ (n=4) are included as control. A WilcoxonMann-Whitney statistical test was performed for each sample. Asterisks indicate statistical significance of the difference: *p value < 0.05, **p value < 0.01, ***p value < 0.001, ****p value <0.0001.
